# HelperFriend, a Serious Game for Promoting Healthy Lifestyle Behaviors in Children: Design and Pilot Study

**DOI:** 10.2196/33412

**Published:** 2022-05-06

**Authors:** Ismael Edrein Espinosa-Curiel, Edgar Efrén Pozas-Bogarin, Maryleidi Hernández-Arvizu, Maria Elena Navarro-Jiménez, Edwin Emeth Delgado-Pérez, Juan Martínez-Miranda, Humberto Pérez-Espinosa

**Affiliations:** 1 Centro de Investigación Científica y de Educación Superior de Ensenada Unidad de Transferencia Tecnológica Tepic Tepic, Nayarit Mexico; 2 Centro de Estudios e Investigaciones en Comportamiento Universidad de Guadalajara Guadalajara, Jalisco Mexico

**Keywords:** serious game, children, education and behavior change, healthy lifestyle behaviors, physical activity, healthy eating, socioemotional wellness

## Abstract

**Background:**

The use of health games is a promising strategy for educating and promoting healthy lifestyle behaviors among children.

**Objective:**

We aimed to describe the design and development of a serious game, called HelperFriend, and evaluate its feasibility, acceptability, and preliminary effects in children in a pilot study. HelperFriend is a vicarious experiential video game designed to promote 3 lifestyle behaviors among young children: physical activity, healthy eating, and socioemotional wellness.

**Methods:**

Participants aged 8 to 11 years were recruited from an elementary school and randomized to receive a healthy lifestyle behavior educational talk (control) or play six 30-minute sessions with HelperFriend (intervention). Assessments were conducted at baseline (T0) and after the intervention (ie, 4 weeks) (T1). The primary outcome was gain in knowledge. The secondary outcomes were intention to conduct healthy behaviors, dietary intake, and player satisfaction.

**Results:**

Knowledge scores of intervention group participants increased from T0 to T1 for physical activity (*t*_14_=2.01, *P*=.03), healthy eating (*t*_14_=3.14, *P*=.003), and socioemotional wellness (*t*_14_=2.75, *P*=.008). In addition, from T0 to T1, the intervention group improved their intention to perform physical activity (*t*_14_=2.82, *P*=.006), healthy eating (*t*_14_=3.44, *P*=.002), and socioemotional wellness (*t*_14_=2.65, *P*=.009); and there was a reduction in their intake of 13 unhealthy foods. HelperFriend was well received by intervention group.

**Conclusions:**

HelperFriend appears to be feasible and acceptable for young children. In addition, this game seems to be a viable tool to help improve the knowledge, the intention to conduct healthy behaviors, and the dietary intake of children; however, a well-powered randomized controlled trial is needed to prove the efficacy of HelperFriend.

## Introduction

### Healthy Lifestyle Behaviors

Unhealthy lifestyle behaviors (eg, physical inactivity, unhealthy diet, and sedentary time) put individuals at high risk of developing several health conditions (eg, dental caries, hypertension, diabetes, cardiopathy, and cancer) and are key drivers of obesity and being overweight [1-3]. In contrast, healthy lifestyle behaviors (eg, physical activity and healthy diet) can provide a general feeling of well-being and are the foundation of disease prevention [4]. Individuals who embrace healthy lifestyle behaviors can withstand health risks linked to disability and illness in later life [4]. Healthy lifestyle behaviors can benefit people of all ages; however, it is crucial to encourage these behaviors from early childhood—when habits are formed—because they are likely to be maintained during adulthood [3,5,6].

Two critical healthy lifestyle behaviors for children are healthy eating and physical activity. Children need to have a correct diet [7], increase the intake of healthy food, decrease the intake of unhealthy food [8], and engage in a minimum of 60 minutes of moderate to vigorous physical activity daily [9]. These behaviors help prevent weight gain in children [10,11] and are included in the strategies to face the dramatic increase in childhood obesity, given that childhood obesity is associated with unhealthy eating and physical inactivity [12,13]. Despite the importance of these behaviors, many children do not perform them. For example, in Mexico, only 43.5% of children meet the recommended intake of fruits [14], 22% of children meet the recommended intake of vegetables [14], and 17.3% of children engage in at least 60 minutes of daily physical activity [15]. The promotion of these behaviors should start when children are young (between 8 and 11 years), because these are the years during which rates of being overweight and obesity increase significantly [15].

Lifestyle interventions should focus on healthy eating and physical activity to have a more significant effect on health [10]. In addition, social and emotional factors should be taken into account when developing lifestyle interventions because these factors affect healthy eating and physical activity [16,17]. Therefore, it is essential that children also learn how to recognize and manage emotions, establish and maintain constructive and healthy relationships, make responsible decisions, and avoid unhealthy social and emotional behaviors associated with eating and physical activity [18,19].

### Serious Games for Health

In recent years, the development of serious games as innovative methods to support health education and treatment initiatives and programs has increased [20]. Serious games integrate engagement and fun elements (eg, stories, levels, rewards, and feedback) with educational and psychological resources and techniques to achieve health outcomes [21]. Serious games for health can include the simulation of real-life situations, collection of information that supports the identification of behaviors, and provision of information and suggestions to guide the process to improve attitudes and behaviors of players [20]. Thus, they offer the possibility to support initiatives to deliver education and health services to populations that currently cannot or do not obtain necessary access to these services due to costs, logistical issues, stigma, or convenience [22]. There are currently serious health games for a wide variety of purposes, such as health education, physical and psychological therapy, and disease self-management [20,23].

Serious games are increasingly being used to encourage children to adopt healthy lifestyle behaviors, leveraging the fact that most children enjoy playing video games [24]. However, there are mixed opinions about this strategy. One concern is related to screen time—screen time is considered to be a risk factor for several health, emotional, and psychological problems in children [25,26]. However, it appears that screen time for playing video games does not represent as high a risk when compared with that for watching television [27]. Another concern is the effectiveness of these games because, while most studies have reported positive effects on obesity-related outcomes (improvement of weight-related parameters, physical activity, or dietary behavior and knowledge), these effects were small [28,29]. In addition, while many games focus on improving health knowledge, this does not necessarily result in behavioral change [28]. Conversely, several studies [30-32] have shown that serious games offer an enormous advantage for health promotion interventions in children.

Video games for promoting healthy lifestyle behaviors in children are aimed to improve knowledge about nutrition, eating habits, and exercise; increase physical activity while playing (exergames); change eating behaviors; or combine several approaches [28,31,33,34]. Nutrition and eating habits–related games focus on the concepts of energy balance [35,36], MyPlate guidelines [37], the 5 macronutrients of foods [38], Mediterranean diet and behavioral moderation [39], healthy and unhealthy nutrition [40,41], and dietary energy density [42]. Despite the importance of psychosocial or psychological aspects of nutrition and eating habits, only one game considered these aspects through the integration of the coping of stress technique [42]. Although positive results were obtained in these studies [35-42], there is still a need to understand the application and limitations of such games as well as how to improve their effectiveness, such as the inclusion of the underlying mechanisms for behavioral change of video games [31] or the integration of psychosocial aspects in video games [28]. In addition, none of these games implemented a vicarious experiential environment that includes behavior change techniques to promote physical activity, promote healthy eating, and address social and emotional issues related to these behaviors in young children.

### Objective and Hypotheses

We aimed to design and develop a motion-controlled serious game for young children (HelperFriend) and evaluate its feasibility, acceptability, and preliminary effects. We hypothesized that children who played the game would demonstrate (1) better knowledge, (2) greater intention to carry out healthy lifestyle behaviors, and (3) improvements in dietary intake and that (4) children would enjoy playing the game.

## Methods

### HelperFriend Video Game

#### Design and Development

HelperFriend was developed by a multidisciplinary team that included nutritionists, psychologists, physical activity experts, human–computer interaction experts, and software engineers based on published design methodology [43]. The methodology included activities from game implementation to evaluation based on 4 essential principles: a procedure-centric approach, expert collaboration, agile development, and low-cost modeling. The knowledge domains of HelperFriend are physical activity, healthy eating, and socioemotional wellness.

HelperFriend integrates experiential and vicarious learning. In experiential learning environment, learners engage in direct experiences to enhance their knowledge, skills, and values through human–environment interaction in a cycle of doing, reflecting, concluding, and trying the learned experience [44]. In vicarious learning, individuals learn from the experiences of others (eg, by observing the choices another person makes and the consequences they have on their health). By observing the behavior of others, individuals can identify difficulties and expectations associated with behaviors and acquire the information and competencies to perform the behavior successfully [45].

In addition, several behavior change techniques [46] were integrated into the game elements to generate an attractive and stimulating environment in which knowledge and healthy lifestyle behaviors are encouraged and reinforced: instruction on how to perform the behavior, providing information about health consequences, behavioral practice, behavioral substitution, incentives and rewards, goal setting, reviewing behaviors goal, monitoring behaviors, providing feedback on behavior, discrepancies between current behaviors and goals, monitoring emotion consequences, and prompts or cues. These behavior change techniques are based on behavioral, cognitive, and social cognitive theories that have ample empirical evidence to demonstrate their usefulness in adopting healthy lifestyles [47-50].

#### Description

**Figure 1:**
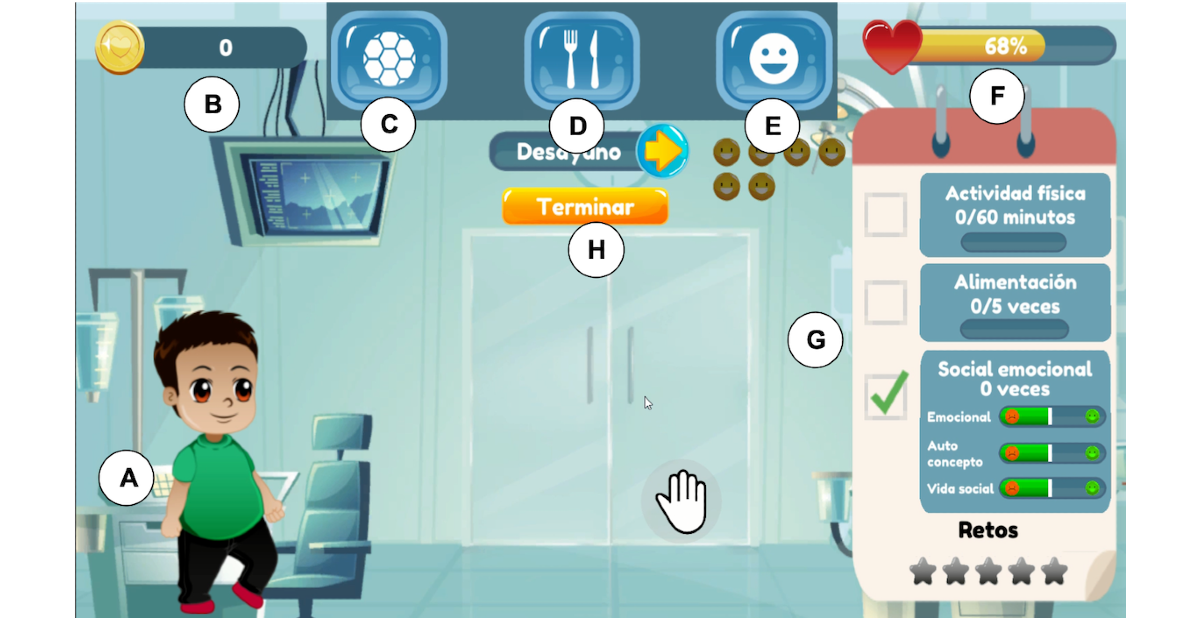
The main screen of HelperFriend: (A) child being cared for, (B) coins score, (C) button to carry out physical activity, (D) button for feeding the child, (E) button to carry out socioemotional activities, (F) health bar of the child, (G) game indicators section, and (H) finish button.

In HelperFriend, the players are secret agents who need to care for a group of children who forgot healthy lifestyle behaviors because a villain chef erased their memory. In each match, the player needs to ensure that one of these children engages in physical activity, eats well, and performs socioemotional activities to improve their health (Figure 1). The children characters continuously interact with the player, expressing their necessities or stating situations for which they need help. Player actions that improve the children's lifestyle behaviors add points. The game session is finished when the player presses the finish button. At the end, the player can earn extra points if their decisions helped the child to meet healthy lifestyle recommendations. The player has full-body interaction to encourage physical activity and improve satisfaction and fun [51]. A video of the game is provided as Multimedia Appendix 1.

#### Modules

##### Overview

Each module increases in difficulty to keep players engaged and having fun until the end of the video game. Modules have 3 components. The education component teaches basic health knowledge. The training component encourages players to practice healthy lifestyle behaviors. The challenge component presents challenging situations in which players have to help the children.

##### Physical Activity

**Figure 2:**
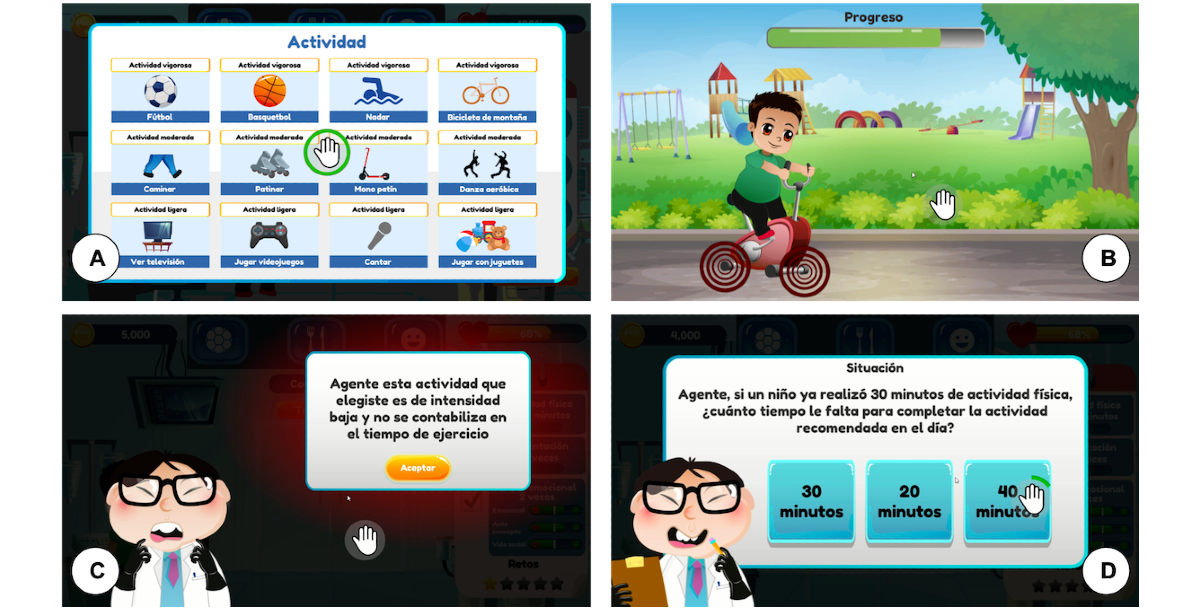
Physical activity screens: (A) screen for selecting a physical activity, (B) child doing physical activity, (C) alert feedback screen because the player selected a sedentary activity, and (D) physical activity situation in which the child needs the player's help.

This module (Figure 2) addresses World Health Organization physical activity recommendations for children and adolescents (aged 5 to 17 years). Children and adolescents should engage in a minimum of 60 minutes of moderate to vigorous physical activity on a daily basis, most of which should consist of aerobic exercise [9]. Engaging in more than 60 minutes of physical activity provides additional health benefits. In addition, vigorous physical activities and muscle and bone strengthening activities each should be incorporated at least 3 days per week.

##### Healthy Eating

**Figure 3:**
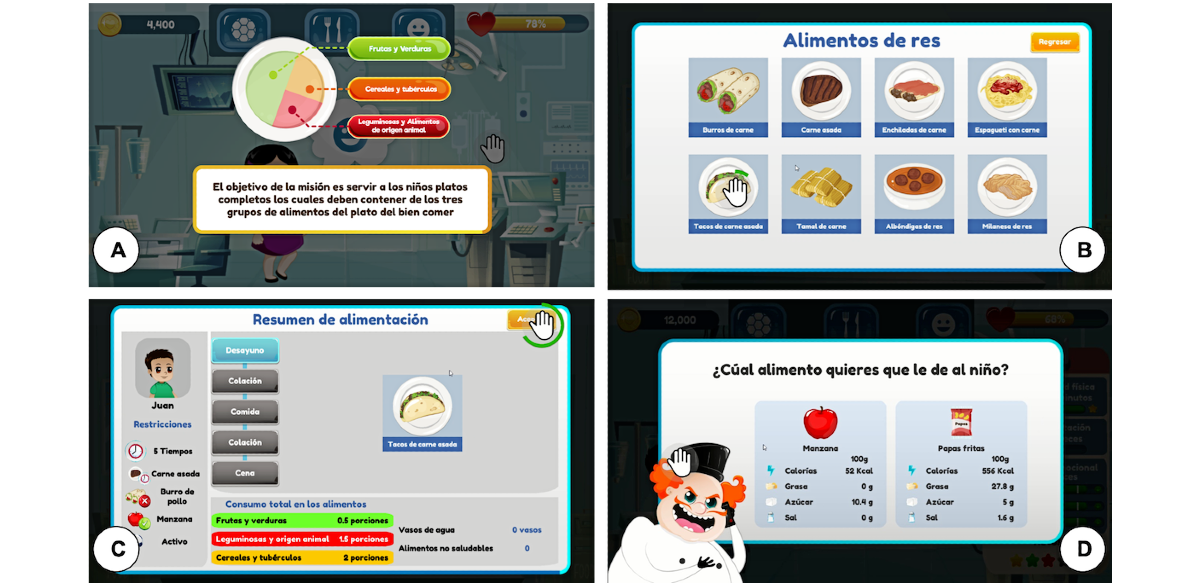
Healthy eating screens: (A) screen teaching player about complete diet, (B) screen for selecting food, (C) feeding information screen, and (D) feeding situation in which the child needs the player's help.

This module (Figure 3) addresses diet. According to the Mexican Official Standard [7], a correct diet for children is one that is complete, balanced, innocuous, suitable, and varied. In addition, this module addresses portion intake and recommendations that children should eat approximately 5 times a day [52], increase water and healthy food (including fruits and vegetables) consumption, and decrease unhealthy food (eg, candies, sweetened cereals, and sugary drinks) consumption [8].

##### Socioemotional

**Figure 4:**
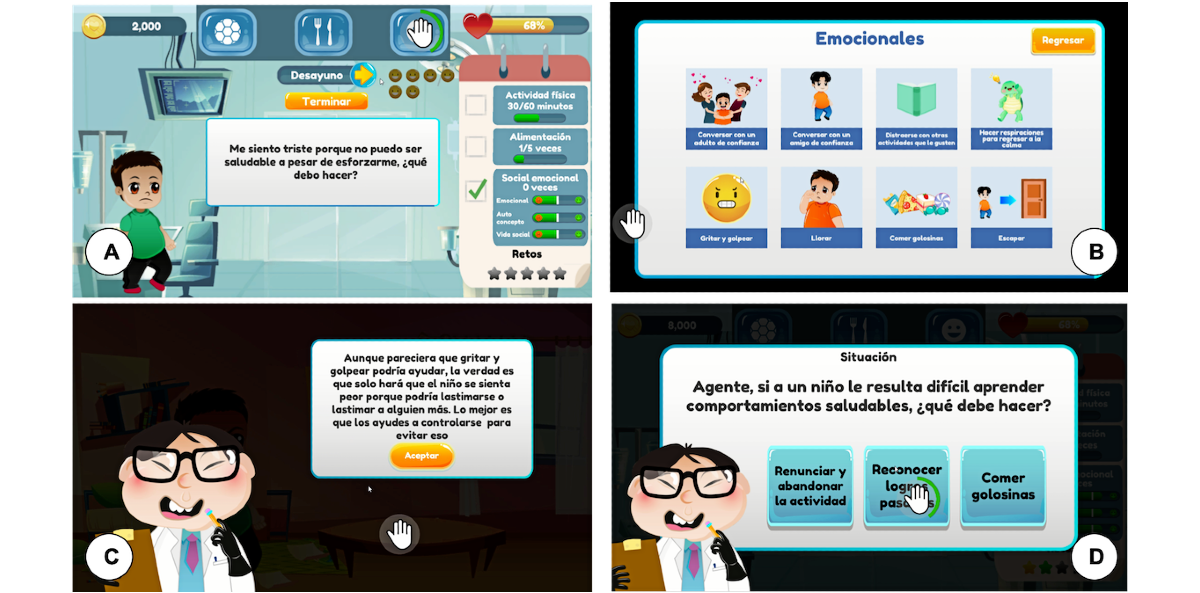
Socioemotional wellness screens: (A) screen where the child shows a socioemotional situation to the player, (B) screen for selecting a socioemotional activity, (C) alert feedback message because the player made an inadequate socioemotional activity choice, and (D) socioemotional situation in which the child needs the player's help.

This module (Figure 4) addresses social and emotional behaviors related to physical activity and healthy eating. Children need to acquire skills to recognize and manage emotions, establish and maintain constructive and healthy relationships, take an interest in the well-being of others, and make responsible decisions [18,19]. Examples of the social and emotional issues included are low motivation to improve exercise and eating habits, emotions associated with eating junk food, and the influence of parents and friends on eating habits and physical activity.

### Intervention

#### Overview

We conducted a parallel randomized controlled pilot trial over 4 weeks between May and June 2019 in an elementary school in Mexico.

#### Ethics

School administrators and teachers gave written permission for the trial to be performed at school facilities. All study procedures were approved by the institutional review board of the *Centro de Investigación Científica y de Educación Superior de Ensenada* (2S.3.1 HUM 2019). No changes occurred to the methods after the beginning of the trial.

#### Participants

Students (n=40) from 3 school groups was considered for this study. Inclusion criteria were being aged 8 to 11 years and not receiving pharmacological treatment. Exclusion criteria were having been diagnosed with or having an ongoing neuropsychiatric disorder, a physical problem (because the game required children to interact through whole-body movements), and obesity treatment in the past 6 months. Written informed consent was obtained from parents of children who expressed interest in participating in the study.

#### Design

Children were randomly allocated to either the control group or the intervention group. The children in the intervention group played HelperFriend during six 30-minute game sessions. All playing sessions were conducted over 21 days. We set up 3 gaming stations in a room; each station contained a PC, a 50-inch screen, a Kinect sensor V2, and the HelperFriend video game. Participants in the control group received only a 45-minute talk about the importance of healthy behaviors, such as engaging in physical activity, eating healthy, and maintaining socioemotional health; no further intervention was applied.

#### Outcome Measures

**Figure 5:**
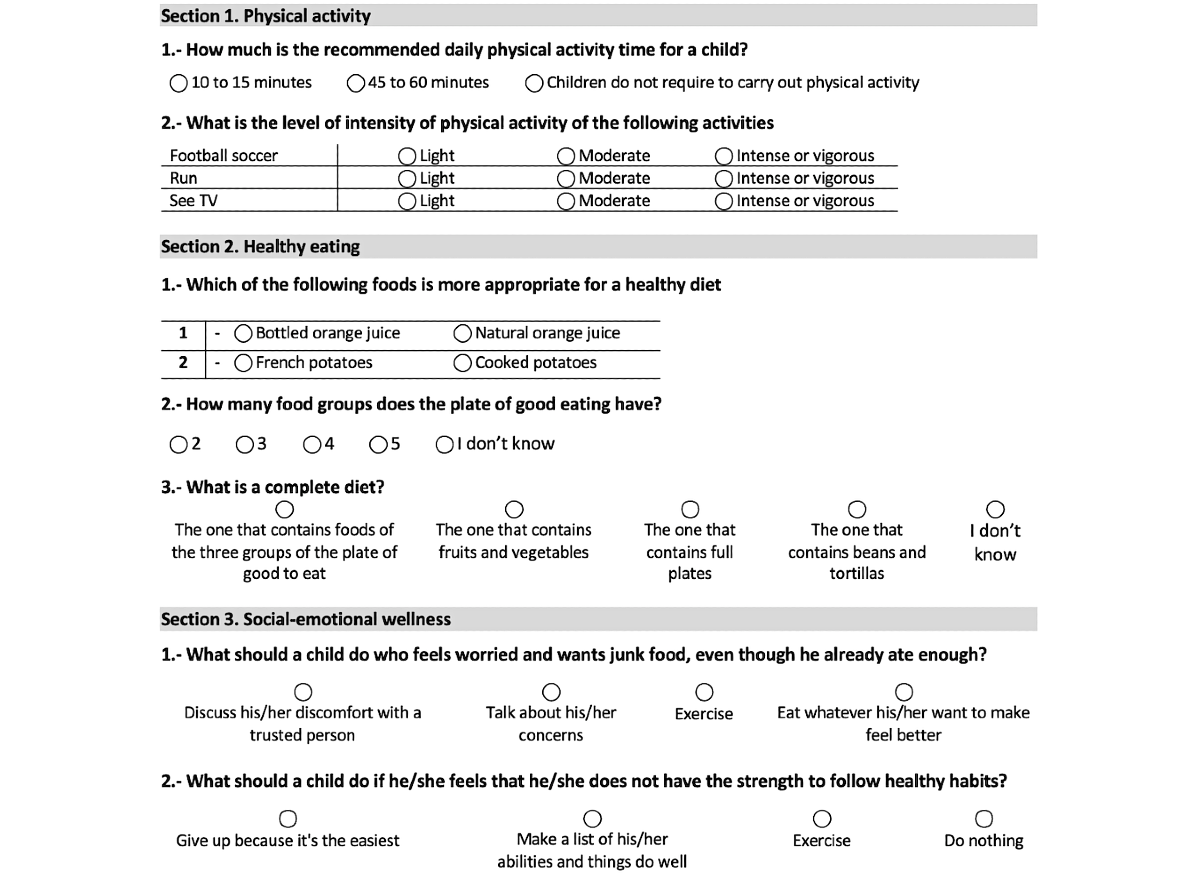
Healthy behaviors knowledge questionnaire example questions.

Outcomes were assessed in both groups the week after being assigned to the groups (T0) and 4 weeks after baseline (T1). The primary outcome was the gain in knowledge measured using a questionnaire (developed by the research group and designed specifically for the serious game). The questionnaire was evaluated in a pilot with 5 children and adapted. The final questionnaire consisted of 82 questions in 3 sections: physical activity (13 questions, each with 3 response options), healthy eating (64 questions, each with 3 to 5 response options in food groups, food portions equivalence, correct diet, and healthy/unhealthy food subsections), and socioemotional wellness (5 questions, each with 4 response options). Figure 5 provides some examples of the questions. Children completed the questionnaire by themselves. The sum of questions that had been appropriately answered for each section was calculated.

Secondary outcomes were intention to conduct healthy behaviors, dietary intake, and player experience satisfaction.

Children’s intention to conduct healthy behaviors was measured using a questionnaire tailored specifically for the serious game. The questionnaire was pilot-tested with 5 children and adapted. The final questionnaire (Multimedia Appendix 2) consisted of 33 questions in 3 sections: physical activity (4 questions), healthy eating and correct diet (24 questions), and socioemotional wellness (5 questions). These questions state everyday situations that children experiment in their daily lives, and they have to decide what action to take to solve the case. Physical activity, healthy eating, and socioemotional wellness questions have 3, 3, and 4 response options, respectively. Questions related to correct diet have 3 response options and use a graphical representation to facilitate children's answers (a previous study [53] used similar graphical questionnaires with children). For each question, there was only 1 appropriate answer, and children completed the questionnaire by themselves. The sum of questions that had been appropriately answered for each section was calculated.

Dietary behavior was measured with a food frequency intake questionnaire [41]. This questionnaire was explicitly designed for the diet of school-age Mexican children and included features of the highest validated food frequency questionnaires. It consists of 78 food items considered to be indicators for healthy and unhealthy eating behaviors. For each item, a 7-point scale, from 0 (never) to 6 (two or more times per day), is used to indicate the frequency that the food is consumed. Each question was scored individually. In order to facilitate the completion of the questionnaire, a facilitator read the questions to the children, who only had to answer with the number of times that they had eaten the food in the past month.

Player experience satisfaction was only assessed in the intervention group (at T1). An adapted version of the Game User Experience Satisfaction Scale [54] was used. The scale consists of 23 questions in 7 domains: playability, narratives, enjoyment, personal gratification, creative freedom, audio aesthetics, and visual aesthetics. We asked participants to specify their agreement level using a 5-point Likert scale, from 1 (totally disagree) to 5 (totally agree). A score was calculated for each domain.

#### Data Analysis

Data were analyzed using SPSS software (version 26; IBM Corp). The statistical significance for all analyses was *P*<.05. Variables are reported as means (with standard deviations) or medians (with interquartile ranges). The normality distribution of interval variables was tested using the Shapiro-Wilk test. For metric data, differences between pretest and posttest were analyzed using 1-tailed paired *t* tests. For nonmetric data, the differences between pretest and posttest were analyzed using Wilcoxon signed-rank sum tests. Differences between groups were analyzed using 1-tailed independent *t* tests. The relationships between subscale items were tested using Cronbach *α*. No power estimation was performed since this was a pilot study.

## Results

### Participant Characteristics

Of 40 children approached for the trial, 27 (68%) children agreed to participate (age: mean 9.9 years, SD 0.9 years; girls: 16/27, 59%; boys: 11/27, 41%). The control group had 12 participants (age: mean 9.8 years, SD 0.62; girls: 7/12, 58%; boys: 4/12, 33%), and the intervention group had 15 participants (age: mean 9.9 years, SD 0.94; girls: 9/15, 60%; boys: 6/15, 40%). We created a game environment where children felt comfortable during game sessions; however, 1 child missed 1 session, and 6 children missed 2 sessions. Participants in the intervention group played an average of 3.1 hours.

### Primary Outcome: Healthy Behaviors Knowledge

Knowledge of intervention group participants increased significantly from T0 to T1 for physical activity (*t*_14_=2.01, *P*=.03), healthy eating (*t*_14_=3.14, *P*=.003), and socioemotional wellness (*t*_14_=2.75, *P*=.008). There were no significant changes in the knowledge of the control group participants from T0 to T1 for physical activity (*t*_11_=0.29, *P*=.39), healthy eating (*t*_11_=–0.64, *P*=.27), and socioemotional wellness (*t*_11_=0.01, *P*=.50). At T1, between-group differences were statistically significant for physical activity (*t*_25_=1.98, *P*=.03), healthy eating (*t*_25_=1.85, *P*=.04), and socioemotional wellness (*t*_25_=1.97, *P*=.03); the intervention group scored higher than the control group for all 3 (Table 1).

**Table 1:** Outcomes of healthy behaviors knowledge and intention to conduct healthy behaviors.

Measure			Items, n		Control group				Intervention group				Between-group postintervention comparison
				Baseline (T0), mean (SD)		Postintervention (T1), mean (SD)	*P* value	Baseline (T0), mean (SD)		Postintervention (T1), mean (SD)	*P* value	*P* value	
**Knowledge**													
	Physical activity	13		4 (1.5)		4.08 (1.6)	.39	4.5 (2)		5.7 (2.4)	.03	.03	
	Healthy eating	64		38.9 (11.9)		38.3 (12.9)	.27	40 (9.2)		45.5 (7.1)	.003	.04	
	Socioemotional wellness	5		1.83 (1.2)		1.83 (1.2)	.50	2.1 (1.2)		2.9 (1.4)	.008	.03	
**Intention to conduct healthy behaviors**													
	Physical activity	4		2.4 (1.4)		2.3 (1.4)	.22	2.13 (1.6)		3.07 (0.8)	.006	.03	
	Healthy eating	24		13.9 (4.3)		14.2 (3.5)	.35	13.5 (3.5)		16.3 (2.2)	.002	.03	
	Socioemotional wellness	5		4.4 (1.2)		4.5 (0.8)	.41	4.6 (0.5)		4.9 (0.25)	.009	.01	

### Secondary Outcomes

#### Intention to Conduct Healthy Behaviors

Intention to perform healthy behaviors in the intervention group increased from T0 to T1 for physical activity (*t*_14_=2.82, *P*=.006), healthy eating (*t*_14_=3.44, *P*=.002), and socioemotional wellness (*t*_14_=2.65, *P*=.009). There were no significant differences in intention to conduct healthy lifestyle behaviors for control group participants for physical activity (*t*_11_=–0.80, *P*=.22), healthy eating (*t*_11_=0.40, *P*=.40), and socioemotional wellness (*t*_11_=0.23, *P*=.41). In addition, differences in T1 intention scores between the intervention group and control group were statistically significant for physical activity (*t*_25_=1.95, *P*=.03), healthy eating (*t*_25_=1.91, *P*=.03), and socioemotional wellness (*t*_25_=2.43, *P*=.01); the intervention group scored higher than the control group for all 3 (Table 1).

#### Food Frequency Intake

Participants in the intervention group reported reduced consumption frequency of ham, sausage, soft drinks, wheat burritos, hamburgers, breaded chicken, sopes, tamales, salt peanuts, sweet cookies, potatoes chips, cake, and sweet soft cakes. Participants in the control group indicated reduced self-reported frequency intake of 5 healthy foods (cantaloupe, carrot, fish soup, fish ceviche, and fresh fruit juice) and 1 unhealthy food (bottled fruit juice) (Table 2).

**Table 2:** Outcomes of food frequency intake.

		Classification	Baseline (T0) score^a^, median (IQR)	Postintervention (T1) score^a^, median (IQR)	*P* value
**Intervention group**					
	Ham	Unhealthy	2 (1.3)	1 (2)	.02
	Sausage	Unhealthy	2 (1.3)	1 (2)	.02
	Soft drinks	Unhealthy	1 (2)	1 (2)	.02
	Wheat burritos	Unhealthy	1 (2)	0 (1)	.03
	Hamburgers	Unhealthy	1 (1)	1 (1)	.01
	Breaded chicken	Unhealthy	0 (1)	0 (0)	.048
	Sopes	Unhealthy	2 (1.3)	0 (1)	.02
	Tamales	Unhealthy	1 (1)	0 (1)	.02
	Salt peanuts	Unhealthy	1 (1.3)	0 (1)	.04
	Sweet cookies	Unhealthy	1 (1.3)	0 (1.25)	.01
	Potato chips	Unhealthy	1 (1)	0 (1)	.03
	Cake	Unhealthy	1 (1.3)	0 (1)	.04
	Sweet soft cakes	Unhealthy	1 (0.3)	0 (1)	.02
**Control group**					
	Cantaloupe	Healthy	1 (1.25)	0 (1)	.047
	Carrot	Healthy	1 (2)	0 (1)	.02
	Fish soup	Healthy	0.5 (2)	0 (0)	.03
	Fish ceviche	Healthy	2 (1.15)	0.5 (1)	.04
	Fresh fruit juice	Healthy	1.5 (2.25)	0 (1)	.02
	Bottle fruit juice	Unhealthy	1.5 (1.25)	0.5 (1)	.04

^a^0 indicated never, 1 indicated one to three times per month, 2 indicated once per week, 3 indicated two to four times per week, 4 indicated five to six times per week, 5 indicated daily, and 6 indicated two or more times per day.

#### Player Video Game Satisfaction

Satisfaction ratings were significantly higher than the neutral value for all domains: playability (*t*_14_=7.04, *P*<.001), narrative (*t*_14_=4.00, *P*<.001), enjoyment (*t*_14_=4.77, *P*<.001), creative freedom (*t*_14_=7.69, *P*<.001), audio aesthetics (*t*_14_=4.33, *P*<.001), personal gratification (*t*_14_=5.99, *P*<.001), and visual aesthetics (*t*_14_=5.12, *P*<.001). Most participants agreed that the game was easy to learn to play and use (14/14, 100%), has a clear history (12/15, 80%), is fun and original (11/15, 73%), has good music (12/15, 80%), has good graphics (12/15, 80%), and made them feel successful when they overcame the game's challenges (13/15, 87%). In addition, most participants wanted to play HelperFriend again (12/15, 80%). All measures obtained Cronbach *α* values ≥.73, except for narrative (Cronbach *α*=.56) (Table 3).

**Table 3:** Player satisfaction questionnaire results.

Measure	Items, n	Cronbach *α* (n=15)	Mean (SD)	Neutral value	*P* value	Lower	Upper
Playability	9	.76	4 (0.5)	3	<.001	2.8	5
Narrative	2	.56	3.9 (0.9)	3	<.001	1.5	5
Enjoyment	3	.73	4 (0.8)	3	<.001	2.7	5
Creative freedom	2	.73	4.1 (0.6)	3	<.001	3	5
Audio aesthetics	3	.90	4.1 (1)	3	<.001	1.7	5
Personal gratification	4	.75	4.2 (0.7)	3	<.001	2.5	5
Visual aesthetics	2	.85	4 (0.8)	3	<.001	2.5	5

## Discussion

### Principal Findings

#### Knowledge

Children in the intervention group significantly improved their knowledge about physical activity (*t*_14_=2.01, *P*=.03), healthy eating (*t*_14_=3.14, *P*=.003), and socioemotional wellness (*t*_14_=2.75, *P*=.008) after gameplay; thus, the first hypothesis was verified. Previous studies have also shown that video games for health can help children improve their understanding of physical activity [55] and healthy eating [35,37-39,41,55]. Other than in a recent study [42], in which stress and stress-coping strategies were included, emotional and social issues related to adopting healthy lifestyle behaviors have not been previously taken into account in interventions with serious games that have targeted healthy eating. We also identified that it is possible to teach this type of issue with serious games. Our results support those of a previous study [55], showing that it is feasible to improve physical activity and healthy eating knowledge together. These promising results may be explained by the experiential and vicarious learning environment of the game in which players observe the behavior of others [45] and engage in direct experiences through doing, reflecting, concluding, and trying the learned experience [44]. Conversely, because improving health knowledge through serious games does not necessarily result in behavioral change [28], we also plan to adjust content and learning strategies in a future version.

#### Intention to Conduct Healthy Behaviors

Second, we hypothesized that the intention to conduct physical activity, healthy eating, and healthy socioemotional behaviors would be higher after the intervention; we also verified this hypothesis. A previous study [40] also showed that video games for health could help children improve dietary and exercise attitudes, but we did not identify any studies on intention to conduct healthy socioemotional behaviors. Improved intention to engage in healthy behaviors may be as a result of integrating multiple behavior change techniques—a cornerstone for efficacy in behavior change interventions [56]. Because a change in intention leads to a small to medium change in behavior [57], these findings help assess the potential impact of video games on children's lifestyle behaviors. The smallest increase in intention was for socioemotional wellness, possibly because the intervention group already scored higher at baseline, which may be associated with the fact that the questionnaire contained simple questions that make it easy to obtain a high score.

#### Food Frequency Intake

The third hypothesis stated that, after the intervention, children's diets would improve. A lower intake frequency was found for 13 unhealthy foods (such as soft drinks, hamburgers, sweet cookies, potatoes chips, and sweet soft cakes). These changes are relevant because Mexican children commonly consume these foods in schools and at home [58] and changes in children's diets and eating habits can promote changes in the whole family [59]. Our findings confirm that games to improve healthy food consumption are beneficial, which has been demonstrated in some earlier studies (eg, [36,39,41]), but not others (eg, [42]). Unlike some previous studies (eg, [35,39]), we only achieved a reduction in the consumption of unhealthy foods. One possible explanation is that providing information or visual images of foods alone is insufficient to increase children's preferences for the intake of healthy foods [60,61]. Instead, repeated exposure to healthy foods is more effective for improving children's preferences [61]—even more so than strategies based on rewards [62]. A future version of HelperFriend should include behavior change techniques (eg, self-monitoring, setting and examining goals, and action planning) that directly support and encourage healthy food intake. Surprisingly, the participants in the control group indicated reduced intake of 5 healthy foods (cantaloupe, carrot, fish soup, fish ceviche, fresh fruit juice) and 1 unhealthy food (bottled fruit juice); however, we did not collect any other information from the control group that could facilitate the interpretation of this result.

#### Game Acceptance and Satisfaction

The fourth hypothesis was also verified; children felt good during gameplay, and game acceptance was high. HelperFriend obtained very positive results on personal gratification, playability, creative freedom, enjoyment, narrative, and visual and audio aesthetics—factors which have been shown to be correlated with and predictors of learning [63]. However, for four specific aspects, there is room for improvement: (1) improving the fit of the difficulty curve of the game to the capacities of the children, (2) simplifying the game flow to foster player autonomy, (3) increasing socioemotional elements, and (4) implementing a daily activity tracking system in the game to make it easier for children to understand the daily activities they have to carry out to have healthy lifestyles. These characteristics could be essential aspects that positively influence the general perception, acceptability, and effectiveness of the game.

### Limitations

First, the results should be cautiously interpreted because a small group of children participated in the study. However, given that we aimed to evaluate the feasibility, acceptability, and preliminary effects of HelperFriend, our findings can offer valuable information in designing health games for children to improve lifestyle behaviors and that consider socioemotional issues. Second, medium- and long-term effects were not examined. Medium- and long-term studies could provide interesting findings since video games, especially those involving physical activity, can become boring quickly [64]. Third, we developed the intention questionnaire because we did not find any available for young children; however, a detailed review would be necessary prior to its use in a full randomized controlled trial. Finally, the frequency of food intake was self-reported. The results could be limited by the known constraints of food frequency questionnaires, such as trouble recalling experiences and over- or understatement of food intake [65]. Nevertheless, food frequency questionnaires are the most frequently used approach because they are easy to use, reliable, and valid. In addition, there is previous evidence that children's self-reported food intake is more accurate than that reported by parents [66].

We plan to conduct a randomized controlled clinical trial with sample size calculation to address some of these limitations. Moreover, we plan to extend the exposure period and conduct repeated exposure to account for medium- and long-term effects. Finally, we plan to improve the intention questionnaire and include another behavioral test (eg, physical activity).

### Conclusions

HelperFriend, a vicarious experiential health game for promoting physical activity, healthy eating, and socioemotional wellness, appears to be feasible and acceptable for young children. Preliminary results suggest that this game improves knowledge about and the intention to conduct healthy lifestyle behaviors and improves dietary intake in children. In future versions of HelperFriend, some game elements should be improved and other behavior change techniques that promote children's intake of healthy foods should be integrated. Given that this was a pilot study with a limited sample size, a well-powered randomized controlled trial is needed to determine the efficacy of HelperFriend.

## Appendix

Multimedia Appendix 1 Video of the game.

Multimedia Appendix 2 Intention to conduct healthy behaviors questionnaire.
