# Supplementary material for: HelperFriend, a Serious Game for Promoting Healthy Lifestyle Behaviors in Children: Design and Pilot Study
Source: JMIR Serious Games. 2022 May 6;10(2):e33412. doi: 10.2196/33412 (PMC9123542; doi:10.2196/33412)
Supplement: Multimedia Appendix 2 [file games_v10i2e33412_app2.pdf]

Questionnaire to measure the intention to perform physical activity, consume a healthy diet and conduct socio emotional well-being activities

(The original version is in Spanish)

ID:

Date: **Start time:** **End time:**

Instructions

This questionnaire asks you about your intentions to conduct physical activity, eat healthy food, and conduct activities for socio-emotional wellness. You must read each question and select the option that you intend to do.

Remember, it is very important that you answer the questions as honestly and sincerely as possible.

Section 1. Physical activity

1. You go to the park with your parents. Consider that you have not done physical activity during the day. From the following available activities, choose the one you would intend to do.

◯ Run ◯ Lay down on the grass ◯ Eat an ice cream

2. It's Friday afternoon, you're at home, and you have free time. Consider that you have not done physical activity during the day. From the following available activities, choose the one you would intend to do.

◯ Sleep ◯ Exercise ◯ Watch a movie

3. You are at your school recess. Consider that you have not done physical activity during the day. From the following available activities, choose the one you would intend to do.

◯ Buy candies ◯ Talk with my friends ◯ Play catch

4. You are visiting your cousins in your uncles' house. Consider that you have not done physical activity during the day. From the following available activities, choose the one you would intend to do.

◯ Play video games ◯ Play ball ◯ Play a board game

Continue on the next page…

Section 2. Healthy eating and correct diet

5. It's time to eat. From the following food, select the one that you would intend to eat.

◯ Fried beans ◯ Cooked beans

6. You are going to have a snack. From the following food, select the one that you would intend to eat.

◯ Potato chips ◯ Natural peanuts

7. You are going to eat. From the following food, select the one that you would intend to eat.

◯ Chicken soup ◯ Fried chicken

8. You are going to have a snack. From the following food, select the one that you would intend to eat.

◯ Vanilla ice cream ◯ Apple

9. You are going to have dinner. From the following food, select the one that you would intend to eat.

◯ Hamburger ◯ Corn quesadillas

10. You are going to have dessert. From the following food, select the one that you would intend to eat.

◯ Chopped fruit ◯ Strawberry-filled chocolate cupcake

11. You are going to have a drink. From the following food, select the one that you would intend to eat.

◯ Fruit water ◯ Milk shake

12. You are going to eat a fruit. From the following food, select the one that you would intend to eat.

◯ Banana ◯ Fried bananas

13. You are going to eat some nuts. From the following food, select the one that you would intend to eat.

◯ Caramelized walnuts ◯ Walnuts

Continue on the next page…

14. You are going to eat at a restaurant with your parents. Which of the following dishes would you propose to eat considering that you must have a complete diet?

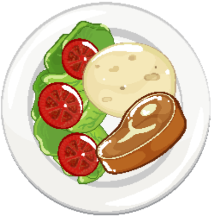

Beef, vegetable salad, and corn tortilla

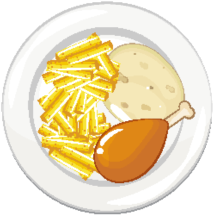

Chicken, potatoes, and corn tortilla

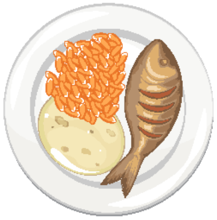

Fish, rice soup ,and corn tortilla

15. A friend invites you to eat at his house. It is important that you consider that you had chilaquiles with chicken for breakfast. Which of the following dishes would you propose to eat considering that you must have a varied diet?

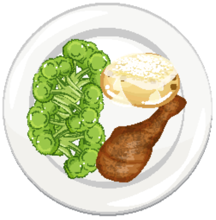

Breaded chicken, cooked broccoli, and baked potato

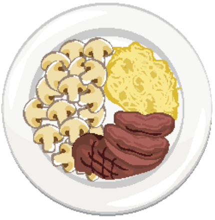

Roast beef, spaghetti, and mushrooms

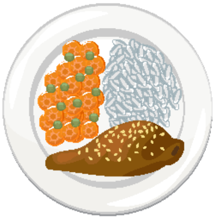

Chicken with mole, steamed vegetables, and white rice

16. You are going to choose your dinner. Consider that you already ate 2 servings of animal foods during the day. Which of the following dishes would you propose to eat considering that you must have a sufficient diet?

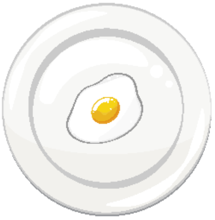

1 serving of fried egg

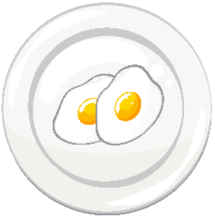

2 servings of fried eggs

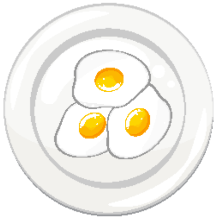

3 servings of fried eggs

Continue on the next page…

17. You are going to have breakfast in the school's dining room. Which dish would you intend to eat considering that you must have a balanced diet?

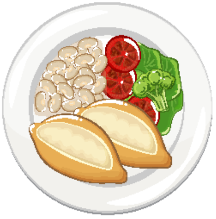

1 serving of beans, 1 serving of vegetable salad, and 2 servings of bread

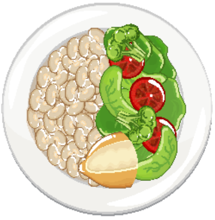

2 servings of beans, 2 servings of vegetable salad, and ½ serving of bread

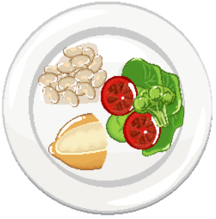

½ serving of beans, ½ serving of vegetable salad, and ½ serving of bread

18. It's time for breakfast, you're going to choose a dish assuming you don't like chard. Which dish would you intend to eat considering that you must have an adequate diet?

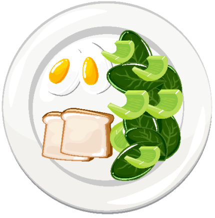

Boiled egg, celery, chard, and box bread

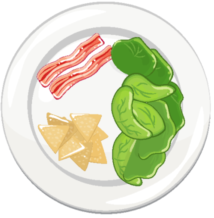

Bacon, chard, and box bread

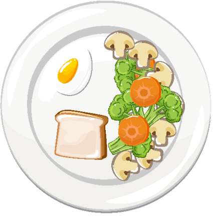

Boiled egg, broccoli, carrot, mushrooms, and box bread

19. Your best friend invites you to eat. Which dish would you intend to eat considering that you must have a complete diet?

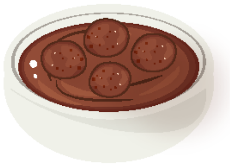

Meatballs

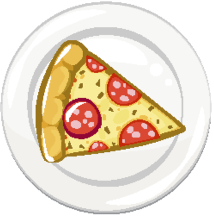

Pepperoni pizza

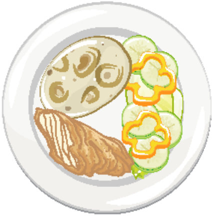

Chicken Milanese, vegetable salad, and flour tortilla

Continue on the next page…

20. A neighbor invites you to dinner at his house. It is important that you consider that you ate roast beef. Which dish would you intend to eat considering that you must have a varied diet?

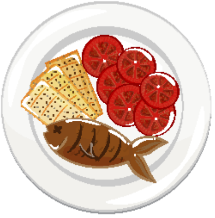

◯ Fried fish, tomato, and crackers

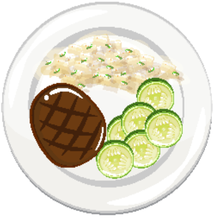

◯ Beef, cucumbers, and mashed potatoes

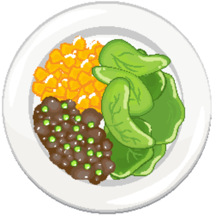

◯ Picadillo, lettuce, and pasta

21. Your mom is preparing your food for dinner. She considers that you already ate 4 servings of vegetables during the day. Which dish would you intend to eat considering that you must have a sufficient diet?

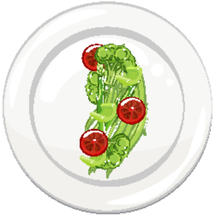

◯ 1 serving of salad

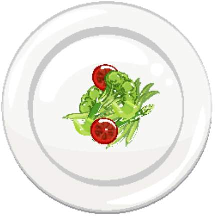

◯ ½ serving of salad

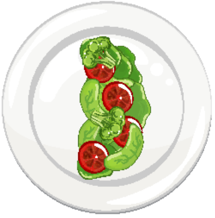

◯ 2 servings of salad

22. You are going to have breakfast with your grandmother. Which dish would you intend to eat considering that you must have a balanced diet?

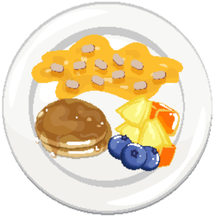

◯ 3 scrambled eggs, ½ serving of chopped fruit, and 2 hotcakes

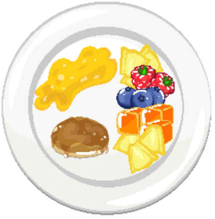

◯ 1 scrambled egg, 1 portion of chopped fruit, and ¾ of hotcake

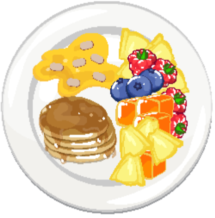

◯ 1 scrambled egg, 2 servings of chopped fruit, and 4 hotcakes

Continue on the next page…

23. It's time to eat, you're going to choose a dish assuming you don't like chickpeas. Which dish would you intend to eat considering that you must have an adequate diet.

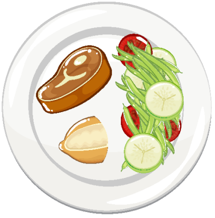

◯ Beef, vegetable salad, and box bread

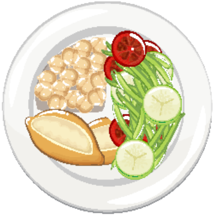

◯ Chickpeas, vegetable salad, and box bread

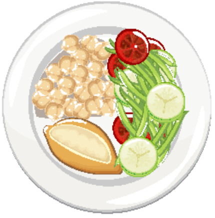

◯ Chickpeas, vegetable salad, and box bread

24. Your cousin invites you to eat. Which dish would you intend to eat considering that you must have a complete diet.

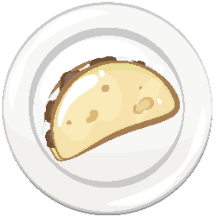

Chicken quesadilla

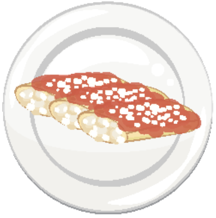

Chicken enchiladas

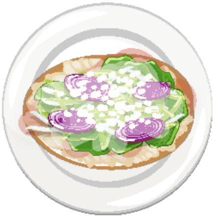

Chicken tostada

25. Your teacher invites you to dinner. It is important that you consider that you ate shrimp cocktail. Which dish would you intend to eat considering that you must have a varied diet.

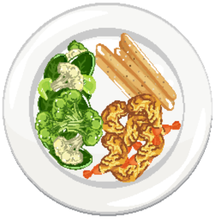

Breaded shrimp, salad, and breadsticks

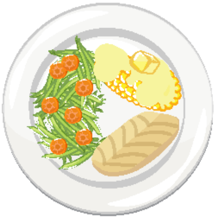

Grilled fish, salad, and corn with butter

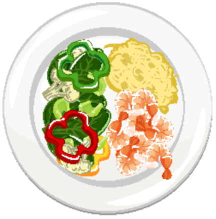

Cooked shrimp, salad, and pasta

Continue on the next page…

26. You are going to accompany your dinner with tortillas. Consider that you already ate 4 servings of cereals during the day. Which dish would you intend to eat considering that you must have a sufficient diet.

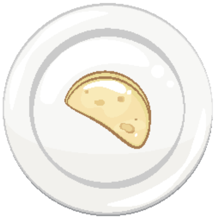

1 portion of corn tortilla

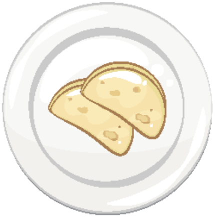

2 portions of corn tortilla

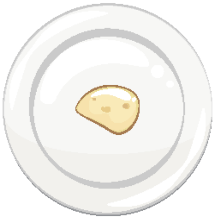

½ portion of corn tortilla

27. You are going to eat at your uncles' house. Which dish one would you intend to eat considering that you must have a balanced diet.

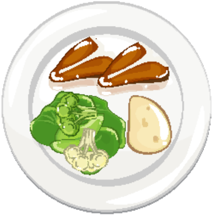

2 portions of meat, ½ portion of salad, and ½ corn tortilla

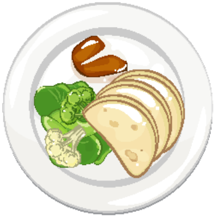

½ portion of meat, ½ portion of salad, and 3 corn tortillas

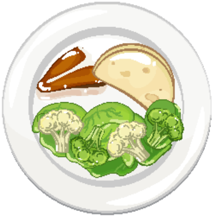

1 portion of meat, 1 portion of salad, and 1 corn tortilla

28. It's dinner time, you're going to choose a dish assuming you don't like sweet potato. Which dish would you intend to eat considering that you must have an adequate diet.

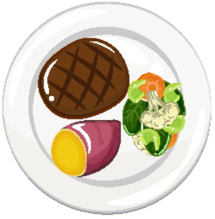

◯ Beef, vegetable salad, and sweet potato puree

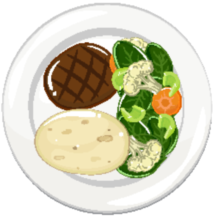

◯ Beef, vegetable salad, and corn tortilla

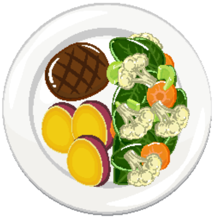

◯ Beef, vegetable salad, and sweet potato puree

Section 3. Socio-emotional wellness

29. You try to have healthy eating habits, but your parents give you a lot of junk food on your birthday. From the following activities available which one would you intend to do?

- ○ Asking your parents to give you more junk food
- ○ Eating all the junk food because it's a gift
- ○ Asking your friends to help you eat the junk food
- ○ Asking your parents to support you in following your healthy eating habits

30. You try to have healthy eating habits, but you feel like you don't have the necessary skills. From the following activities available which one would you intend to do?

- ○ Eating sweets to cheer yourself up
- ○ Striving to eat healthy food
- ○ Exercise
- ○ Give up because it's the easiest

31. You have not managed to have healthy habits, and you want to do something to change the situation. From the following activities available which one would you intend to do?

- ○ Rest and sleep
- ○ Talk about your concerns with a friend
- ○ Strive to eat healthy foods and exercise
- ○ Do nothing, it doesn't matter to be healthy

32. You try to have healthy eating habits and do physical activity, but you feel worried and all the time you want to eat. From the following activities available which one would you intend to do?

- ○ Talk about your concerns with a friend
- ○ Discuss your concerns with a trusted adult
- ○ Do nothing, it doesn't matter to be healthy
- ○ Eating to feel better

33. You try to have healthy eating habits, but you feel tired and get angry easily. You want to do something to change the situation. From the following activities available which one would you intend to do?

- ○ Rest and sleep
- ○ Yelling and hitting things
- ○ Talk about your concerns with a friend
- ○ Discuss your concerns with a trusted adult

Very good ... You finished the questionnaire. Thanks for participating!
